# Exploring the impact of Cabotegravir‐Rilpivirine long‐acting on weight gain, body composition and quality of life in adults living with HIV

**DOI:** 10.1111/hiv.70202

**Published:** 2026-01-28

**Authors:** Andrea De Vito, Andrea Marongiu, Antonella Cano, Mariangela Puci, Agnese Colpani, Susanna Maria Nuvoli, Maria Grazia Catte, Giulia Moi, Maria Antonietta Deledda, Sergio Uzzau, Giovanni Sotgiu, Franca Deriu, Angela Spanu, Giordano Madeddu

**Affiliations:** ^1^ Unit of Infectious Diseases, Department of Medicine, Surgery, and Pharmacy University of Sassari Sassari Italy; ^2^ PhD School in Biomedical Science, Biomedical Science Department University of Sassari Sassari Italy; ^3^ Unit of Nuclear Medicine, Department of Medicine, Surgery and Pharmacy University of Sassari Sassari Italy; ^4^ Department of Biomedical Sciences University of Sassari Sassari Italy; ^5^ University Hospital of Sassari, Sassari (AOUSS) Sassari Italy; ^6^ Clinical Epidemiology and Medical Statistics Unit, Department of Medicine, Surgery and Pharmacy University of Sassari Sassari Italy; ^7^ Unit of Infectious Diseases, Department of Medicine Ospedale San Francesco Nuoro Italy; ^8^ Unit of Microbiology and Virology University Hospital of Sassari Sassari Italy; ^9^ Unit of Endocrinology, Nutritional and Metabolic Disorders University Hospital of Sassari, Sassari (AOUSS) Sassari Italy

**Keywords:** antiretroviral therapy, bioelectrical impedance analysis, body composition, Cabotegravir, dual‐energy X‐ray absorptiometry, HIV, long‐acting injectables, metabolic changes, Rilpivirine

## Abstract

**Introduction:**

Long‐acting injectable antiretroviral therapy (ART) with Cabotegravir (CAB) and Rilpivirine (RPV) offers an alternative to daily oral regimens, improving adherence and patient satisfaction. However, its impact on body composition and metabolism remains underexplored.

**Methods:**

We conducted a prospective cohort study involving 29 people with HIV initiating CAB + RPV LA at a single centre in Italy. Body composition was assessed using dual‐energy X‐ray absorptiometry (DXA) and bioelectrical impedance analysis (BIA) at baseline, 24 and 48 weeks. Anthropometrics, laboratory parameters and patient‐reported outcomes were also collected. Statistical comparisons across time points were performed using paired tests (*p* < 0.05 considered significant).

**Results:**

At 48 weeks, weight and BMI remained stable. Waist circumference significantly decreased (median 97 (IQR 91–102) to 94 (IQR 89–98) cm, *p* = 0.026), with no significant change in total fat percentage or visceral adipose tissue. A modest but statistically significant increase in trunk/limb fat ratio (mean 1.19 (SD 0.39) to 1.25 (SD 0.41), *p* = 0.035). Lean mass and muscle function were unchanged. BIA findings confirmed stable fat mass and body water compartments. Virologic suppression was maintained in all participants throughout follow‐up. High‐density lipoprotein (HDL) cholesterol increased significantly, accompanied by a rise in total cholesterol, while low‐density lipoprotein (LDL) cholesterol, triglycerides and the total cholesterol/HDL ratio remained stable. Serum creatinine significantly decreased, mainly among individuals switching from bictegravir‐ or dolutegravir‐based regimens. Glycaemia, insulin, HOMA‐IR, Metabolic Score for Insulin Resistance (METS‐IR), liver enzymes and hepatic steatosis and fibrosis indices remained stable. Adverse events, mostly injection‐site reactions, decreased over time. Only one participant discontinued treatment. Treatment satisfaction improved throughout the study.

**Conclusion:**

CAB + RPV LA was not associated with significant weight gain, clinically relevant changes in body composition or adverse metabolic effects over 48 weeks. Virologic suppression was maintained, renal laboratory parameters improved in prior INSTI users and treatment was well tolerated with increasing satisfaction. These findings support CAB + RPV LA as a safe, effective and metabolically neutral alternative to daily oral ART.

## INTRODUCTION

The global burden of human immunodeficiency virus (HIV) continues to challenge public health, with millions of people worldwide living with this condition [1]. Antiretroviral therapy (ART) has been the cornerstone of HIV management since the 1990s, reducing mortality and improving the quality of life [2, 3]. The landscape of ART has evolved significantly over the years, with the development of more effective, safer and more tolerable drugs [3]. However, adherence to daily oral regimens can affect long‐term success, favouring the development of innovative drug delivery systems, including long‐acting (LA) injectable formulations, which promise to transform HIV treatment by improving adherence and patient satisfaction [4].

In particular, the first LA injectable formulation approved by the Food and Drug Administration (FDA) and European Medicines Agency (EMA) is the combination of Cabotegravir (CAB) and Rilpivirine (RPV), which maintain virologic suppression following monthly or every two months administration [5, 6, 7, 8, 9, 10, 11]. The most frequently reported adverse events (AEs) were injection‐site reactions (ISRs) such as pain, which, although common, typically resolve within a few days. Serious AEs (SAEs) are rare, and the discontinuation rate is low [5, 7, 11]. Despite these issues, the LA regimen presents a high level of treatment satisfaction reported by participants [6, 7, 8, 12, 13, 14].

Recent studies have raised concerns regarding weight gain and alterations in body composition associated with integrase strand transfer inhibitors (INSTIs), particularly first‐generation agents such as raltegravir and some second‐generation agents such as dolutegravir (DTG), with more heterogeneous findings for bictegravir (BIC) [15, 16]. These effects have been linked to increases in total fat mass, visceral adipose tissue (VAT) and trunk‐to‐limb fat redistribution, as reported in both clinical trials and real‐world cohorts evaluating body composition through DXA or CT imaging [17, 18]. Increased VAT has been strongly associated with cardiometabolic complications and a higher risk of coronary artery disease (CAD) in people with and without HIV [19, 20, 21, 22].

The mechanisms underlying these changes are not fully understood, but proposed pathways include alterations in adipocyte differentiation, mitochondrial function and insulin signalling [23, 24, 25]. Hormonal pathways have also been hypothesised to modulate weight gain during INSTI‐based therapy. Prior studies suggest possible interactions with the oestrogen–melanocortin axis, which may contribute to sex‐specific adipose accumulation [26, 27]. However, evidence for direct effects on androgen or testosterone regulation remains limited, and findings are inconsistent across clinical cohorts [28, 29].

Regarding CAB + RPV LA there is an important gap in the literature on their effect on weight gain and metabolic changes. The only available data came from the SOLAR trial [30] and did not show weight changes between CAB + RPV LA and Tenofovir Alafenamide (TAF)/emtricitabine (FTC)/BIC. The present study aims to evaluate changes in body composition using a dual‐energy x‐ray absorptiometry (DXA) scan and bioelectrical impedance analysis (BIA) in patients treated with CAB plus RPV every two months.

## MATERIALS AND METHODS

We conducted a prospective cohort study, including people with HIV followed at the Infectious Diseases Unit in Sassari, who initiated treatment with CAB + RPV LA. We consecutively enrolled all individuals who visited the centre and opted to start LA treatment.

Inclusion criteria were (i) HIV‐RNA measured within a maximum of 4 months before starting the treatment <50 copies/mL; (ii) having at least 18 years old; (iii) undergoing BIA and DXA; (iv) signature of informed consent. Exclusion criteria were (i) any documented NNRTI‐ or INSTI‐associated resistance mutation identified in the most recent or in any previous genotype resistance test (GRT); (ii) any history of virological failure while receiving an NNRTI‐ or INSTI‐based regimen; (iii) people with a BMI lower than 15 or higher than 40; (iv) requirement for haemodialysis; (v) Child‐Pugh Score C; (vi) previous SAEs related to NNRTI and/or INSTI and (vii) positive HBsAg.

### DXA

A total body DXA scan was performed on all participants the day of the first CAB + RPV LA administration and at 24 (±4) and 48 (±4) weeks.

Total body soft tissue composition was measured by DXA with DPX‐L, Lunar Corporation (Madison, WI, USA), acquisition and analysis software 4.6. Markers used in this study for trunk and lower limbs that defined regions of interest were those indicated by the manufacturer.

**FIGURE 1:**
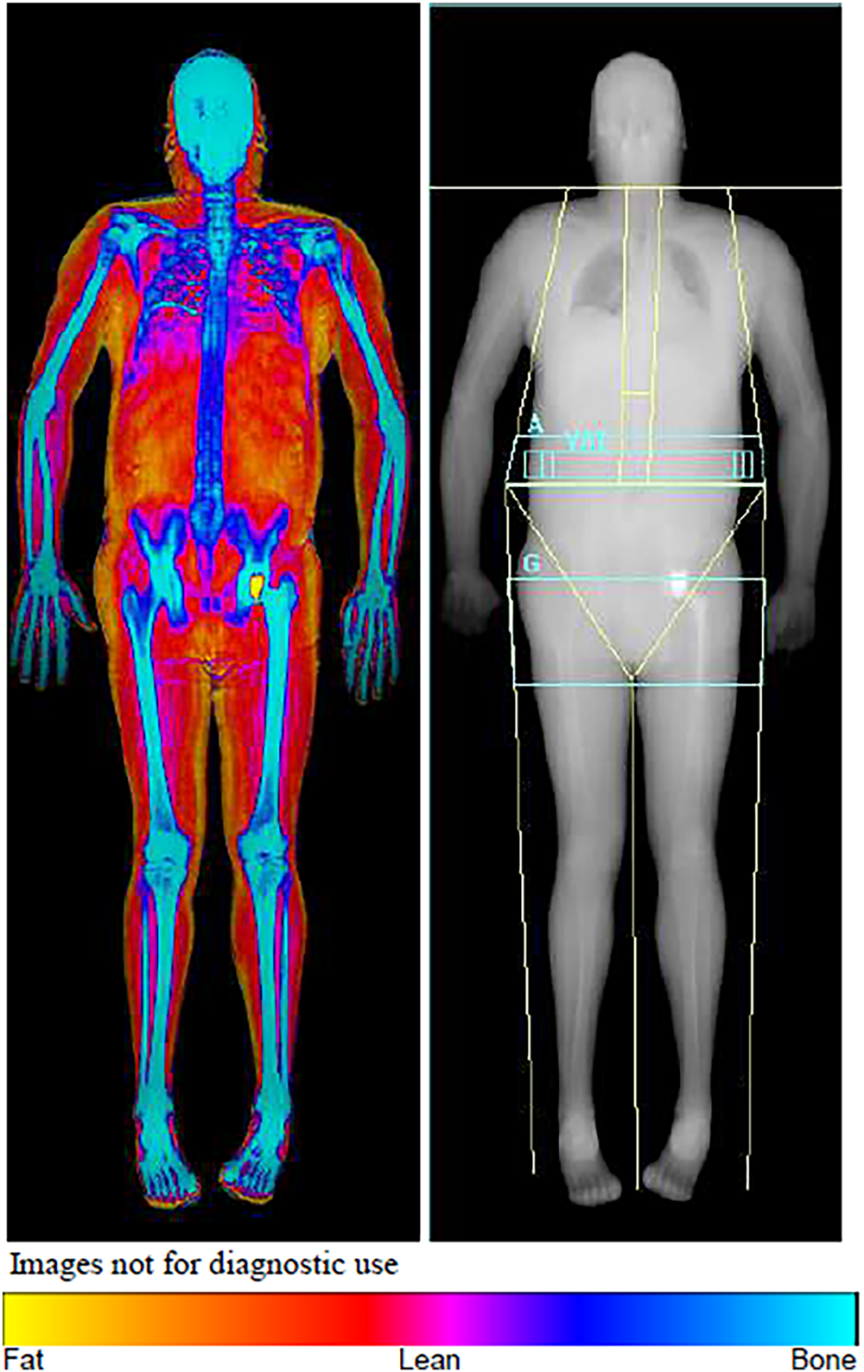
DXA scan of a participant.

Total fat mass percentage (TFM%), the Visceral Adipose Tissue (VAT) mass expressed in kg, android/gynoid ratio (A/G ratio), trunk/leg ratio, trunk/limb ratio and the lean/height ratio were evaluated (Figure 1).

### BIA

BIA was performed on all participants the day of the first CAB + RPV LA administration and at 24 (±4) and 48 (±4) weeks using the Bioelectrical Vector Analyser (BIA101, Akern, Italy) and analysed using Sofware BodygramPlus.

The participants had to be fasting and were tested supine, after a 5‐minute rest, on a non‐conducting surface with their arms slightly abducted away from their trunk and the legs separated. Absolute and percent body cell mass (BCM), fat mass (FM), fat‐free mass (FFM), total body water (TBW), extracellular water (ECW) and intracellular water (ICW), skeletal muscle mass (SMM) and appendicular skeletal muscle mass (ASMM) were estimated and compared at different time points.

### Muscle strength assessment

Handgrip strength was assessed using a handgrip dynamometer (Model G200, Biometrics Ltd., Newport, United Kingdom) across three trials, with a one‐minute rest interval between each trial. The best and mean values obtained were used for analysis. Muscle quality index, as best HGS (kg)/ASMM (kg) ratio, was calculated and used as a variable for the statistical analysis.

### Clinical history, anthropometric measurements and blood tests

Before the first visit, the clinical history of all participants was reviewed to assess eligibility for starting CAB + RPV LA treatment, collecting personal data and habits, the HIV history and other medical history.

Anthropometric measurements and blood samples were collected at the first administration of CAB + RPV LA, at 24 (±4) and 48 (±4) weeks.

Anthropometric measurements included height, weight, body mass index (BMI), waist and abdominal circumferences and blood pressure.

HIV‐RNA, lymphocyte subpopulations (CD4, CD8, CD4/CD8 ratio), aspartate transaminase (AST), alanine transaminase (ALT), gamma‐GT, alkaline phosphatase, creatinine, urea, proteinuria, total cholesterol, low‐density lipoprotein (LDL), high‐density lipoprotein (HDL), triglycerides, fasting insulin levels, TSH, fT3, fT4, total testosterone were measured. Additionally, progesterone and estradiol were tested in females.

Cardiovascular risk was assessed using the SCORE‐2 algorithm for participants aged 40–69 years, the SCORE‐2 Older Persons (SCORE‐2OP) model for those aged ≥70 years and the SCORE‐2 Diabetes tool for individuals with a diagnosis of diabetes. Insulin resistance indices were calculated only in participants without known diabetes. Specifically, we used the Homeostatic Model Assessment for Insulin Resistance (HOMA‐IR) and the Metabolic Score for Insulin Resistance (METS‐IR). Hepatic steatosis was evaluated through the fatty liver index (FLI) and Steatosis‐Associated Fibrosis Estimator (SAFE) score. Liver fibrosis risk was assessed using the Fibrosis‐4 (FIB‐4) index. All indices were calculated at baseline, week 24 and week 48.

### Questionnaires

Five questionnaires were administered.

- International Physical Activity Questionnaire (IPAQ) (each visit)
- 5‐level EQ‐5D version (EQ‐5D‐5L) (before treatment, after 4, 24 and 48 weeks).
- HIV Treatment Satisfaction Questionnaire (HIVTSQ) (before treatment change, at 24 and 48 weeks).
- Food diary: 11‐page questionnaire (each visit).
- A questionnaire to investigate AEs (each visit).

### Ethical approval

The Institutional Review Board and the Clinical Research Ethics Committee approved the study protocol (ID: PG2021/17857/2021). Study procedures were carried out in accordance with the Declaration of Helsinki. Written informed consent was obtained from each participant before inclusion.

### Statistical analysis

Data were summarized with absolute numbers and percentages for the qualitative variables and as mean ± standard deviation (SD) or median and interquartile range (IQR) according to the normality of distribution of continuous variables. The normality of distribution was assessed using the Shapiro–Wilk test.

For baseline comparisons between independent groups, qualitative variables were analysed using the Chi‐square test or Fisher's exact test, as appropriate. For longitudinal paired qualitative variables, changes across time points were evaluated using the McNemar test for dichotomous variables and the McNemar–Bowker test for categorical variables with more than two categories. For the continuous variables, changes in the different time points were analysed using the paired Student‐T test or Wilcoxon paired test according to the normality of distribution. A *p‐value* was considered significant when <0.05. Data analysis was performed using STATA17 software.

## RESULTS

We included 29 individuals, 11 assigned females at birth and 18 assigned males at birth, with a median age of 51.7 (48.3–58.4) years. Participants had a long history of HIV treatment, with a median (IQR) length of ART of 12.3 (5.9–23.8) years. All participants started CAB + RPV LA without any oral lead‐in (Table 1). One participant discontinued treatment after 24 weeks due to a persistent headache. Symptoms resolved within one week after resuming the previous regimen (Darunavir/cobicistat monotherapy). This participant had a long‐standing HIV history (36 years) and had previously switched multiple antiretroviral regimens because of recurrent AEs.

**TABLE 1:** Characteristics of people with HIV starting long‐acting treatment with Cabotegravir plus Rilpivirine.

Characteristics	Participants
Age (years), mean (SD)	51.4 (11.8)
Italian, *n* (%)	28 (96.6)
Assigned female at birth, *n* (%)	11 (37.9)
Route of HIV infection	
Heterosexual intercourse	12 (41.4)
MSM	11 (37.9)
PWID	6 (20.7)
Smoking	
Yes	12 (41.4)
Former	9 (31.0)
No	8 (27.6)
Chronic conditions, *n* (%)	
Diabetes	1 (3.4)
Hypertension	10 (34.5)
Dyslipidaemia	22 (75.9)
Depression	3 (10.3)
Anxiety	5 (17.2)
BMI mean (SD)	26.1 (3.5)
Normal weight, *n* (%)	14 (48.3)
Overweight, *n* (%)	11 (37.9)
Obesity I grade, *n* (%)	4 (13.8)
Length of HIV infection (years) median (IQR)	14.1 (6.7–27.3)
Nadir CD4 (cells/mL), median (IQR)	250 (90–528)
Zenith HIV (copies/mL), median (IQR)	78′000 (33′000–250′000)
History of AIDS, *n* (%)	4 (13.8)
Duration of ART (years), median (IQR)	12.3 (5.9–23.8)
History of NNRTI treatment, *n* (%)	18 (62.1)
History of INSTI treatment, *n* (%)	24 (82.8)
Last treatment, *n* (%)	
3TC/DTG	11 (37.9)
TAF/FTC/BIC	9 (31.0)
TAF/FTC/RPV	6 (20.7)
RPV/DTG	2 (6.9)
DRV/c	1 (3.5)

Abbreviations: 3TC, lamivudine; ART, antiretroviral treatment; BIC, bictegravir; DTG, dolutegravir; FTC, emtricitabine; INSTI, Integrase Strand Transfer Inhibitor; IQR, interquartile range; MSM, men who have sex with men; NNRTI, non‐nucleoside retro‐transcriptase inhibitors; PWID, people who injected drugs; RPV, rilpivirine; SD, standard deviation; TAF, tenofovir alafenamide fumarate.

Body weight and BMI did not show significant changes (mean weight 72.7 (SD 13.3) kg to 73.2 (SD 13.3) kg, *p*: 0.382; BMI: 26.2 (SD 3.5) to 26.4 (SD 3.6), *p*: 0.767). Waist circumference significantly decreased (median 97 (IQR 91.5–102) cm to 94 (IQR 89–98) cm, *p*: 0.026), whereas abdominal circumference remained unchanged.

DXA showed no significant variations in total mean body fat, VAT mass, android/gynoid ratio or trunk/leg ratio. However, the trunk/limb fat ratio significantly increased (1.19 (SD 0.39) to 1.25 (SD 0.41), *p*: 0.035). Bioelectrical impedance analysis (BIA) confirmed these findings, with no significant changes observed in fat mass, fat‐free mass or muscle mass (Table 2).

**TABLE 2:** Anthropometric, DXA and BIA changes at 24 and 48 weeks in people with HIV who started long‐acting treatment with Cabotegravir plus Rilpivirine.

Variables	Baseline versus 24 weeks Mean (SD) or median (IQR)^a^		*p*‐value	Baseline versus 48 weeks Median (IQR)^b^		*p*‐value
Body Weight (kg)	72.5 (13.1)	72.2 (13.2)	0.426	72.7 (13.3)	73.2 (13.3)	0.382
BMI	26.1 (3.5)	26.0 (3.5)	0.412	26.2 (3.5)	26.4 (3.6)	0.350
BMI categories, *n* (%)			0.368			0.223
Normal weight	14 (48.3)	13 (44.8)		13 (46.4)	10 (35.7)	
Overweight	11 (37.9)	13 (44.8)		11 (39.3)	14 (50.0)	
Obesity	4 (13.8)	3 (10.3)		4 (14.3)	4 (14.3)	
Waist circumference (cm)	97 (91–102)	94 (89–98)	0.017	97 (91.5–102)	94 (89–98)	0.026
Abdominal circumference (cm)	92 (82–99)	92 (82–98)	0.834	91 (82–100)	91 (82.5–99.5)	0.836
Handgrip Kg (dominant hand)	34.5 (11.1)	33.7 (11.5)	0.453	34.1 (11.4)	32.7 (12.9)	0.203
Muscle quality index	1.26 (0.17)	1.25 (0.28)	0.769	1.25 (0.17)	1.21 (0.26)	0.369
DXA scan						
Total fat (%)	32.6 (7.4)	31.6 (6.8)	0.097	32.8 (7.3)	31.9 (7.0)	0.182
Vat mass (g)	648.2 (305.4)	628.5 (311.7)	0.471	658.0 (306.3)	641.4 (296.4)	0.520
Android/gynoid ratio	1.13 (0.28)	1.13 (0.29)	0.855	1.13 (0.28)	1.13 (0.29)	0.649
Trunk/leg ratio	1.09 (0.31)	1.11 (0.30)	0.380	1.10 (0.31)	1.08 (0.28)	0.309
Trunk/limb ratio	1.18 (0.39)	1.24 (0.41)	0.035	1.19 (0.39)	1.25 (0.41)	0.035
Lean/height	16.3 (2.5)	16.4 (2.4)	0.574	16.3 (2.5)	16.5 (2.6)	0.130
BIA						
Fat mass (%)	23.4 (7.8)	23.4 (7.6)	0.981	23.6 (7.8)	23.7 (7.6)	0.867
Fat mass (Kg)	17.1 (6.8)	17.1 (6.7)	0.896	16.4 (12.6–21.8)	16.7 (12.5–23.5)	0.396
Fat‐free mass (%)	76.6 (7.8)	76.6 (7.6)	0.981	76.4 (7.8)	76.3 (7.6)	0.867
Fat‐free mass (Kg)	56.2 (45.3–64.9)	57.8 (45.7–62.7)	0.471	57.9 (45.1–65.3)	58.4 (44.5–64.4)	0.192
Total body water (%)	56.1 (5.7)	56.1 (5.6)	0.992	55.9 (5.8)	55.9 (5.6)	0.947
Total body water (Kg)	41.3 (33.4–47.7)	42.2 (33.5–45.9)	0.491	42.4 (33.1–48.0)	42.8 (32.6–47.3)	0.187
Extracellular water (%)	43.9 (2.7)	44.0 (2.7)	0.852	43.9 (2.8)	43.9 (2.9)	0.818
Extracellular water (Kg)	17.7 (2.9)	17.7 (2.7)	0.601	18.2 (15.8–19.9)	18.2 (15.6–20.8)	0.195
Body cell mass (%)	11.2 (1.8)	11.2 (1.8)	0.619	10.8 (9.7–12.8)	10.8 (9.7–12.8)	0.682
Body cell mass (Kg)	30.7 (23.3–36.5)	32.7 (23.9–35.6)	0.491	32.0 (23.2–36.8)	32.7 (23.8–36.2	0.319
Muscle mass (%)	37.1 (6.4)	37.1 (6.2)	0.991	36.9 (6.5)	36.8 (6.2)	0.765
Muscle mass (Kg)	28.1 (20.1–33.1)	28.8 (20.6–32.0)	0.568	28.2 (20.0–33.1)	29.0 (20.1–33.1)	0.448
Fat mass index	6.3 (2.6)	6.3 (2.5)	0.862	6.4 (2.6)	6.4 (2.5)	0.721
Phase angle (°)	6.4 (0.6)	6.4 (0.6)	0.715	6.4 (6.1–7.0)	6.3 (6.0–7.1)	0.751
Free fat mass index	20.1 (2.5)	20.0 (2.4)	0.566	20.1 (2.5)	20.2 (2.5)	0.288
Basal metabolic, Kcal	1641 (1427–1808)	1697 (1443–1782)	0.495	1679 (1425–1816)	1696 (1439–1798)	0.646

Abbreviations: BIA, bioelectrical impedance analysis; BMI, body mass index; DXA, dual‐energy X‐ray absorptiometry; IQR, interquartile range; Kg, kilograms; SD, standard deviation; VAT, visceral adipose tissue.

^a^ Data on 29 people.

^b^ Data on 28 people.

Virological suppression was maintained in all individuals, and no virological failures occurred during the study period. Changes in immunological and laboratory parameters are summarized in Table 3. CD4 and CD8 counts showed no significant changes, and the CD4/CD8 ratio remained stable. Total cholesterol increased, whereas HDL cholesterol significantly improved (*p* = 0.011). Cardiovascular risk, assessed via SCORE‐2 or SCORE‐2OP based on age, remained stable. Renal function improved with median serum creatinine decreasing from 0.92 (IQR 0.84–0.99) to 0.83 (IQR 0.77–0.90) mg/dL (*p* < 0.001). Sensitivity analyses stratified by prior antiretroviral exposure showed that this reduction occurred almost exclusively in participants switching from DTG or BIC‐based regimens, in whom median creatinine decreased from 0.87 (IQR 0.75–1.08) at baseline to 0.83 (IQR 0.74–0.92) at 24 weeks (*p =* 0.002*)* and 0.77 (IQR 0.69–0.91) at 48 weeks (*p* < 0.001). In contrast, participants without prior INSTI exposure showed no significant variation, with median values of 0.92 (IQR 0.80–1.17) at baseline, 0.91 (IQR 0.74–1.06) at 24 weeks and 0.95 (IQR 0.71–1.10) at 48 weeks.

**TABLE 3:** Change in blood tests in people with HIV who started long‐acting treatment with Cabotegravir plus Rilpivirine.

Variables	Baseline versus 24 weeks Mean (SD) or median (IQR)^a^		*p*‐value	Baseline versus 48 weeks Mean (SD) or Median (IQR)^b^		*p*‐value
CD4 (cell/mm^3^)	865 (619–1080)	834 (665–959)	0.302	876 (624–1084)	831 (620–1044)	0.897
CD8 (cell/mm^3^)	803 (672–1126)	796 (731–1223)	0.825	798 (669–1131)	935 (636–1100)	0.618
CD4/CD8 (cell/mm^3^)	0.86 (0.73–1.36)	0.81 (0.67–1.24)	0.831	0.87 (0.73–1.42)	0.92 (0.70–1.42)	0.350
Total cholesterol (mg/dL)	184.0 (28.8)	189.8 (37.5)	0.312	183.6 (29.3)	192.0 (32.9)	0.041
LDL (mg/dL)	110.1 (26.8)	110.4 (33.6)	0.940	109.8 (27.3)	112.7 (25.5)	0.317
HDL (mg/dL)	49 (44–62)	55 (47–67)	0.007	51 (46–56)	57.5 (52.5–62.5)	0.011
Total cholesterol/HDL	3.52 (3.14–3.90	3.29 (2.80–4.17)	0.190	3.53 (3.16–3.98)	3.37 (3.00–4.03)	0.522
Triglycerides (mg/dL)	107.6 (47.3)	113.9 (69.2)	0.516	97.5 (72–130)	83 (69.5–131.5)	0.415
Creatinine (mg/dL)	0.91 (0.19)	0.85 (0.17)	<0.001	0.92 (0.84–0.99)	0.83 (0.77–0.90)	<0.001
Fasting glycaemia, (mg/dL)	85 (77–100)	87 (80–94)	0.902	85 (77.5–100)	90.5 (84–101)	0.415
AST (U/L)	19 (17–26)	22 (17–27)	0.794	19 (16.5–26.5)	21.5 (19–26.5)	0.586
ALT (U/L)	22 (15–33)	22 (14–31)	0.904	21.5 (14.5–33.5)	21.5 (16.5–31.5)	0.783
GGT	22 (15–35)	22 (16–29)	0.561	22 (15–34.5)	21 (16–38)	0.111
Alkaline phosphate	66 (59–81)	67 (56–79)	0.933	66 (57–78.5)	66.5 (55.5–79.5)	0.962
Albuminaemia (g/dL)	4 (3.8–4.2)	4.1 (4–4.3)	0.025	4.05 (3.85–4.25)	4.1 (3.95–4.3)	0.352
Platelets (10^9^/L)	221 (178–253)	220 (194–270)	0.333	228 (182–254.5)	219.5 (192–252)	0.625
TSH (cell/mm^3^)	1.82 (1.17–2.33)	1.80 (1.28–2.41)	0.340	1.90 (1.11–2.58)	1.83 (1.27–2.80)	0.091
fT3	3.53 (0.33)	3.66 (0.36)	0.045	3.52 (3.30–3.73)	3.52 (3.39–3.77)	0.271
fT4	1.08 (0.15)	1.19 (0.18)	<0.001	1.09 (0.15)	1.14 (0.14)	0.035
Insulin fasting (cell/mm^3^)	8.6 (5.3–12.8)	9.0 (6.0–11.6)	0.338	7.9 (5.15–12.4)	9.15 (6.1–12.5)	0.087
Total testosterone	458.8 (24.0–573.9)	415 (31.0–588.0)	0.094	458.3 (23.05–566.7)	362.2 (24.4–464.5)	0.005
Male total testosterone^c^	559.4 (500.0–644.5)	567 (450.1–638.0)	0.090	559.4 (500.0–623.5)	452.3 (405.5–537.0)	0.013
Female total testosterone^d^	22.1 (22.0–29.9)	20.6 (18.8–32.6)	0.734	22.1 (21.9–29.9)	22.9 (18.7–26.11)	0.413
Female progesterone^d^	0.39 (0.24–2.73)	0.54 (0.31–0.71)	0.638	0.39 (0.24–2.73)	0.40 (0.29–0.48)	1.000
Female oestrogen^d^	11.8 (11.8–61.7)	19.4 (11.8–133.7)	0.258	11.8 (11.8–61.7)	13.3 (6.1–21.9)	0.172
Score2‐2OP‐2 Diabetes	5.0 (2.9–6.8)	5.0 (2.7–6.4)	0.232	5.0 (2.8–6.8)	4.3 (2.9–5.7)	0.140
HOMA index	1.47 (1.00–2.68)	1.74 (1.19–2.38)	0.350	1.40 (0.94–2.72)	1.89 (1.26–2.77)	0.090
HOMA index >2	13 (46.4)	11 (39.3)	0.687	12 (44.4)	12 (44.4)	1.00
METS‐IR Score	37.6 (7.4)	36.9 (7.6)	0.289	37.8 (7.4)	37.6 (7.5)	0.759
METS‐IR > 50.39	2 (7.1)	0 (0)	0.500	2 (7.4)	2 (7.4)	1.000
Fatty liver index	24.6 (13.1–64.5)	25.6 (13.6–66.7)	0.624	23.9 (12.5–66.0)	22.7 (13.5–61.6)	0.902
Fatty liver index			0.368			0.563
Low risk (<30)	15 (51.7)	17 (58.6)		15 (53.6)	15 (53.6)	
Indeterminate risk (30–59)	6 (20.7)	4 (13.8)		5 (17.9)	6 (21.4)	
High risk (≥60)	8 (27.6)	8 (27.6)		8 (28.6)	7 (25.0)	
FIB‐4	1.13 (0.91–1.32)	1.06 (0.77–1.46)	0.701	1.12 (0.89–1.31)	1.04 (0.77–1.44)	0.552
FIB‐4			0.375			0.375
<1.45	24 (82.8)	21 (72.4)		24 (85.7)	21 (75.0)	
1.45–3.25	5 (17.2)	8 (27.6)		4 (14.3)	7 (25.0)	
>3.25	0	0		0	0	
SAFE Score	−42.2 (89.6)	−36.4 (85.8)	0.454	−45.0 (89.9)	−43.7 (88.0)	0.885
SAFE Score			1.000			1.000
Low risk (<0)	20 (69.0)	20 (69.0)		20 (71.4)	20 (71.4)	
Indeterminate risk (0–99)	8 (27.6)	8 (27.6)		7 (25.0)	7 (25.0)	
High risk (≥100)	1 (3.4)	1 (3.4)		1 (3.6)	1 (3.6)	

Abbreviations: fT3, free‐triiodothyronine; fT4, free‐thyroxine; HDL, high‐density lipoprotein; IQR, interquartile range; LDL, low‐density lipoprotein; SD, standard deviation; TSH, thyroid‐stimulating hormone.

^a^ Data on 29 people.

^b^ Data on 28 people.

^c^ Data on 18 males.

^d^ Data on 11 females.

Fasting glycaemia remained stable during follow‐up, and no cases of new‐onset diabetes were observed. Insulin concentrations did not vary significantly, and HOMA‐IR values remained unchanged. The proportion of individuals with HOMA‐IR >2 did not change over time (44.4% at both baseline and week 48). Similarly, METS‐IR scores remained stable, with no shift in the proportion of participants exceeding the metabolic‐risk threshold.

Liver enzymes showed no significant changes throughout follow‐up. AST, ALT, GGT and alkaline phosphatase remained within normal ranges, and albumin concentrations were stable, indicating preserved hepatic function. The FLI remained unchanged, with a stable distribution of low‐, intermediate‐ and high‐risk categories. Also, the FIB‐4 score and SAFE score remained stable. Overall, these findings indicate no progression of hepatic steatosis or fibrosis and no biochemical evidence of hepatotoxicity during treatment with CAB + RPV LA.

fT4 increased slightly but significantly (mean 1.09 (SD 0.15) to 1.14 (SD 0.14) ng/dL, *p*: 0.035). Total testosterone declined significantly (median 458.3 (IQR 22.1–566.7) to 362.2 (IQR 24.4–464.5) ng/dL, *p*: 0.005). No significant changes were observed in progesterone or oestrogen concentrations among female participants.

A sensitivity analysis was conducted according to prior regimen (TAF‐based vs. non‐TAF), presence of baseline dyslipidaemia and gender. No differences were observed in weight, BMI, lipid changes or metabolic and liver parameters across prior regimen and gender (Tables S1 and S2). Among participants without dyslipidaemia (*n* = 7), total cholesterol and LDL increased at week 48, while HDL and triglycerides and total cholesterol/HDL ratio remained stable (Tables S4 and S5). In contrast, individuals with dyslipidaemia (*n* = 22) experienced an increase in HDL at both 24 and 48 weeks (*p* = 0.014 and *p* = 0.016), while total cholesterol and LDL showed moderate increases at week 48, without significant changes in triglycerides, total cholesterol/HDL ratio or SCORE2‐OP.

## TOLERABILITY AND QUALITY OF LIFE

A high percentage of participants experienced at least one AE. After the first injection, 86.7% reported at least one AE, with a significant decrease with subsequent injections (*p* < 0.001). By the seventh injection, only 24.1% reported experiencing any AE (Figure S1). For the ISR and muscular pain, a decrease over time was recorded (Figures S2 and S3). Fever was reported by 13.3% after the first injection, but it decreased to 3.4% by the seventh. Gastrointestinal disturbances had the highest incidence at the second injection (23.3%), but they declined to 3.4% by the seventh injection (Figure S4).

AEs related to the nervous system decreased over time. Insomnia was reported by 23.3% after the first injection but disappeared by the fourth injection (Figure S5). Only one patient discontinued the therapy due to persistent headaches after 24 weeks.

Despite the AEs, a statistically significant increase in the level of satisfaction with the new therapy was observed (Table S6).

## DISCUSSION

The role of CAB + RPV LA on weight gain, body composition, metabolic markers and quality of life was evaluated in people with HIV over 48 weeks. No significant weight gain or adverse changes in body composition were recorded. Furthermore, patients reported high satisfaction, even though AEs were reported with the first injections.

Concerns regarding weight gain have been reported predominantly with second‐generation integrase inhibitors, particularly DTG and BIC, whereas first‐generation agents such as raltegravir and elvitegravir have generally demonstrated a more neutral effect on body weight [15, 31, 32]. Studies have shown that weight gain and adipose redistribution associated with INSTIs, particularly DTG, contribute to an increased risk of cardiovascular and metabolic complications [33, 34]. Notably, such effects are more pronounced in women and Black individuals, following insulin sensitivity and adipose tissue metabolism changes [35]. In contrast, CAB, showing a different pharmacokinetics profile, appears to exhibit a more favourable metabolic impact. Evidence from HPTN 077 and similar studies reveals that CAB shows minimal effect on weight and metabolic parameters in both people with and without HIV, likely associated with its stable release, which avoids the peak‐to‐trough fluctuations contributing to metabolic stress [36].

Our findings showed non‐significant changes in weight and BMI, which align with findings from other trials, such as FLAIR, ATLAS and SOLAR [5, 37]. Waist circumference and fat distribution indices, including waist‐to‐height and waist‐to‐hip ratios, remained stable. Although waist circumference showed a small reduction while abdominal circumference did not change, this discrepancy is unlikely to reflect muscle loss or visceral fat modification, as DXA and BIA measurements showed no decrease in lean mass and no increase in VAT. Instead, it may reflect minor variations in external anthropometric measurements rather than true alterations in body composition. CAB + RPV LA, therefore, does not appear to affect central adiposity or fat distribution, even though some INSTIs and thymidine analogues are historically associated with central fat gain and peripheral lipoatrophy [38, 39].

Long‐term studies, such as those with the Modena HIV Metabolic Cohort, showed progressive fat accumulation across different body regions following extended ART, with notable sex differences [40, 41]: Women exposed to TAF and integrase inhibitors experienced increased trunk fat, whereas men gained fat in limb areas [15, 40, 42]. Fat accumulation can vary depending on the ART class, demographics and duration of therapy. Although our study could not assess long‐term changes, the lack of significant fat redistribution in the short term supports the assumption that CAB + RPV LA may have a less pronounced impact on adiposity.

Mechanisms underlying ART‐associated changes in body composition involve both direct pharmacologic effects on adipose tissue and indirect immune‐mediated effects [43]. INSTIs, including CAB, may interact with pathways linked to glucose metabolism, lipid handling and inflammatory responses, potentially influencing adipose tissue accumulation [27, 43, 44, 45, 46]. CAB LA formulation achieves steady plasma concentrations without fluctuations in the daily dosing. Additionally, markers of gut barrier dysfunction, such as lipopolysaccharide‐binding protein, were linked to adiposity in people with HIV [47].

We observed a modest increase in HDL‐c at 48 weeks, accompanied by a parallel rise in total cholesterol, while the total cholesterol/HDL ratio remained unchanged. No participant was receiving TDF prior to switching to CAB + RPV LA, excluding TDF withdrawal as a potential explanation for the HDL increase. The overall pattern is consistent with previous research indicating that CAB + RPV LA does not adversely affect lipid profiles. For instance, Adachi et al. reported similar improvements in HDL‐c and stability of the cholesterol/HDL ratio after switching to CAB + RPV LA [48]. In our sensitivity analyses, participants with dyslipidaemia at baseline exhibited increases in total cholesterol, LDL and HDL at 48 weeks, but without deterioration of the cholesterol/HDL ratio or SCORE‐2 cardiovascular risk. In individuals without dyslipidaemia (*n* = 6), isolated increases in LDL and total cholesterol were observed, although the small sample size limits firm conclusions. Taken together, these findings support a generally neutral lipid effect of CAB + RPV LA, with small absolute changes that did not translate into increased cardiovascular risk.

The reduction in serum creatinine observed after switching to CAB + RPV LA was primarily driven by individuals previously treated with DTG or BIC, consistent with the known reversible increase in creatinine caused by OCT2/MATE1 inhibition by these INSTIs [49, 50, 51]. Participants without prior INSTI exposure showed no significant change, suggesting that the decrease reflects withdrawal of transporter inhibition rather than a direct renal effect of CAB + RPV LA.

In our cohort, total testosterone showed a modest decline over time, but values remained within the normal physiological range for all participants. When analysed separately by sex, this reduction was observed only in men, while oestrogen and progesterone levels (measured exclusively in women) remained stable. Neither CAB nor RPV is known to affect androgen pathways, and no corresponding changes in lean mass or metabolic parameters were detected. These findings suggest that the observed fluctuation in testosterone is unlikely to reflect a drug‐related hormonal effect and may instead be attributable to physiologic variation, assay variability or individual clinical factors. Larger studies are needed to confirm whether this observation has any clinical relevance.

The high treatment satisfaction we described aligns with results from the SOLAR and ATLAS‐2 M studies, where participants who transitioned from daily oral BIC/FTC/TAF to CAB + RPV LA increased their treatment satisfaction scores [11, 37].

Several limitations should be considered. First, the sample size was small, and the study was monocentric, which may limit the generalizability of the results and their applicability to more diverse populations. Second, the follow‐up period was relatively short, and although DXA and BIA provide valuable insights into body composition, they may not detect subtle changes over a short timeframe or within small cohorts; longer observational periods would be necessary to evaluate long‐term metabolic effects and confirm the stability of the findings. However, data from the literature have consistently shown that the majority of ART‐associated weight gain—particularly with INSTI‐ and TAF‐based regimens—occurs early, typically within the first 24 to 48 weeks after initiation or switch [25, 52]. After this period, weight gain generally plateaus. Therefore, if CAB + RPV LA had a similar propensity to induce weight or fat accumulation, we would expect to observe such changes within our follow‐up window. The absence of clinically relevant weight or body composition variations at both 24 and 48 weeks supports the hypothesis that CAB + RPV LA exerts a more neutral metabolic effect compared with other contemporary ART options.

Further research with larger, more diverse populations and extended follow‐up is needed to confirm our findings. Assessment of long‐term cardiovascular outcomes associated with CAB + RPV LA could provide insights for clinical management.

## CONCLUSION

CAB + RPV LA therapy in people with HIV was not associated with significant weight gain or adverse changes in body composition over 48 weeks. The small reduction in waist circumference, in the absence of changes in abdominal measurements, lean mass or VAT, suggests no clinically relevant modification in body composition. Metabolic parameters remained stable, and renal function improved. Despite initial AEs, especially ISRs, a high treatment satisfaction and quality of life improvement were reported. These findings support the use of CAB + RPV LA as an effective and well‐tolerated treatment option that does not negatively impact weight or metabolic health in the short term and could be a valuable option for patients needing alternatives to daily oral regimens.

## AUTHOR CONTRIBUTIONS

Andrea De Vito: conceptualization, methodology, investigation, formal analysis, writing – original draft, project administration and visualization. Andrea Marongiu: investigation, data curation and writing – review & editing. Antonella Cano: investigation, data curation, writing – review and editing. Mariangela Puci: formal analysis, data curation, validation and writing – review and editing. Agnese Colpani: data curation, investigation and writing – review and editing. Susanna Maria Nuvoli: investigation, data curation, writing – review and editing. Maria Grazia Catte: investigation, data curation and writing – review and editing. Maria Antonietta Deledda: investigation, data curation, writing – review and editing. Sergio Uzzau: investigation, supervision, writing – review and editing. Giovanni Sotgiu: validation, formal analysis, supervision, writing – review & editing. Franca Deriu: investigation, supervision, writing – review and editing. Angela Spanu: investigation, supervision, writing – review and editing. Giordano Madeddu: supervision, conceptualization, writing – review and editing and project administration.

## FUNDING INFORMATION

This research received no external funding. All activities were conducted as part of routine academic and clinical operations at the University of Sassari. No pharmaceutical or industry sponsors were involved in the design, conduct or publication of this study.

## CONFLICT OF INTEREST STATEMENT

The authors declare no conflicts of interest related to this work. No author has received personal payments, speaking fees or other financial incentives from manufacturers of CAB or RPV.

## ETHICS STATEMENT

The study protocol was reviewed and approved by the Institutional Review Board and Clinical Research Ethics Committee of the University of Sassari (Protocol ID: PG2021/17857/2021). All study procedures were conducted in accordance with the Declaration of Helsinki and Good Clinical Practice guidelines.

## PATIENT CONSENT STATEMENT

Written informed consent was obtained from all participants prior to enrolment in the study, including consent for the use of anonymized data in research and publication.

## PERMISSION TO REPRODUCE MATERIAL FROM OTHER SOURCES

No material from other sources requiring permission was reproduced in this manuscript. All figures and tables are original and created by the authors.

## Supporting information


**Data S1:** Supporting Information

## Data Availability

The data that support the findings of this study are available on request from the corresponding author. The data are not publicly available due to privacy or ethical restrictions.
