# Supplementary material for: Exploring the impact of Cabotegravir‐Rilpivirine long‐acting on weight gain, body composition and quality of life in adults living with HIV
Source: HIV Med. 2026 Jan 28;27(5):727–39. doi: 10.1111/hiv.70202 (PMC13139997; doi:10.1111/hiv.70202)
Supplement: Supplementary file 1 — Data S1: Supporting Information [file HIV-27-727-s001.docx]

Table S1. Baseline characteristics of people with HIV starting CAB+RPV LA by previous regimens TAF or no-TAF based

| **Characteristics** | **Overall** | **People receiving TAF (n=15)** | **People not receiving TAF (n=14)** | **p-value** |
| --- | --- | --- | --- | --- |
| Age (years), mean (SD) | 51.4 (11.8) | 46.8 (11.7) | 56.4 (9.9) | 0.025 |
| Italian, n(%) | 28 (96.6) | 15 (100) | 13 (92.9) | 0.483 |
| Assigned female at birth, n(%) | 11 (37.9) | 4 (26.7) | 7 (50.0) | 0.196 |
| Route of HIV infection |  |  |  | 0.178 |
| Heterosexual intercourse | 12 (41.4) | 7 (46.7) | 5 (35.7) |  |
| MSM | 11 (37.9) | 7 (46.7) | 4 (28.6) |  |
| PWID | 6 (20.7) | 1 (6.6) | 5 (35.7) |  |
| Smoking |  |  |  | 0.294 |
| Yes | 12 (41.4) | 4 (26.7) | 8 (57.1) |  |
| Former | 9 (31.0) | 6 (40.0) | 3 (21.4) |  |
| No | 8 (27.6) | 5 (33.3) | 3 (21.4) |  |
| Chronic conditions, n(%) |  |  |  |  |
| Diabetes | 1 (3.4) | 0 (0) | 1 (7.1) | 0.483 |
| Hypertension | 10 (34.5) | 3 (20.0) | 7 (50.0) | 0.095 |
| Dyslipidemia | 22 (75.9) | 10 (66.7) | 12 (85.7) | 0.224 |
| Depression | 3 (10.3) | 0 (0) | 3 (21.4) | 0.100 |
| Anxiety | 5 (17.2) | 1 (6.7) | 4 (28.6) | 0.143 |
| Length of HIV infection (years) median (IQR) | 14.1 (6.7-27.3) | 8.3 (5.1-13.7) | 29.1 (18.1-34.7) | <0.001 |
| Nadir CD4 (cells/mL), median (IQR) | 250 (90-528) | 420 (106-575) | 197 (52-325) | 0.046 |
| Zenith HIV (copies/mL), median (IQR) | 78’000 (33’000-250’000) | 33’800 (17’000-130’000) | 200’000 (54’000-570’000) | 0.011 |
| History of AIDS, n(%) | 4 (13.8) | 1 (6.7) | 3 (21.4) | 0.151 |
| Duration of ART (years), median (IQR) | 12.3 (5.9-23.8) | 8.3 (5.1-12.8) | 23.9 (13.7-29.3) | 0.003 |
| History of NNRTI treatment, n(%) | 18 (62.1) | 8 (53.3) | 10 (71.4) | 0.316 |
| History of INSTI treatment, n(%) | 24 (82.8) | 10 (66.7) | 14 (100) | 0.025 |
| Last treatment, n(%) |  |  |  |  |
| 3TC/DTG | 11 (37.9) | - | 11 (78.6) |  |
| TAF/FTC/BIC | 9 (31.0) | 9 (60.0) | - |  |
| TAF/FTC/RPV | 6 (20.7) | 6 (40.0) | - |  |
| RPV/DTG | 2 (6.8) | - | 2 (14.2) |  |
| DRV/c | 1 (3.4) | - | 1 (7.1) |  |
| SD: standard deviation; MSM: men who have sex with men; PWID: people who injected drugs; IQR: interquartile range; ART: antiretroviral treatment; NNRTI: non-nucleoside retro-transcriptase inhibitors; INSTI: Integrase Strand Transfer Inhibitor; 3TC: lamivudine; DTG: dolutegravir; TAF: tenofovir alafenamide fumarate; FTC: emtricitabine; BIC: bictegravir; RPV: rilpivirine; DRV/c: darunavir/cobicistat | | | | |

Table S2. Changes in anthropometric values and lipid profile according to TAF or no-TAF as previous regimens

|  | **TAF 0 (n= 14)** | **TAF 1 (n= 15)** | **p-value** |
| --- | --- | --- | --- |
| Mean (SD) weight baseline | 69.1 (10.2) | 75.7 (14.9) | 0.18 |
| Mean (SD) weight 6-months | 68.8 (10.7) | 75.5 (14.9) | 0.18 |
| Mean (SD) weight 12-months | 69.3 (9.8) | 76.6 (15.3) | 0.15 |
| Mean (SD) BMI baseline | 26.1 (2.9) | 26.1 (4.1) | 0.23 |
| Mean (SD) BMI 6-months | 26.0 (3.0) | 26.0 (4.0) | 0.99 |
| Mean (SD) BMI 12-months | 26.3 (2.9) | 26.4 (4.3) | 0.93 |
| Median (IQR) flank baseline | 96 (90-102) | 98 (91-102) | 0.67 |
| Mean (SD) flank 6-months | 94 (6.0) | 94.2 (5.9) | 0.93 |
| Median (IQR) flank 12-months | 94 (89-97) | 94 (92-99) | 0.42 |
| Mean (SD) abdomen baseline | 90.2 (9.4) | 90.5 (11.6) | 0.45 |
| Mean (SD) abdomen 6-months | 91 (9.0) | 89.7 (11.7) | 0.75 |
| Mean (SD) abdomen 12-months | 90.5 (9.7) | 90.3 (11.3) | 0.96 |
| Mean (SD) LDL baseline | 101.9 (32.3) | 117.8 (18.3) | 0.11 |
| Mean (SD) LDL 6-months | 109.1 (41.3) | 111.7 (26.1) | 0.84 |
| Mean (SD) LDL 12-months | 116.1 (41.6) | 125.1 (22.3) | 0.47 |
| Mean (SD) HDL baseline | 45.9 (13.3) | 56.7 (10.0) | **0.02** |
| Mean (SD) HDL 6-months | 50.9 (13.1) | 60.1 (13.9) | 0.08 |
| Mean (SD) HDL 12-months | 53.2 (17.2) | 58.9 (10.7) | 0.29 |
| Mean (SD) C-tot baseline | 174.7 (35.9) | 192.8 (17.3) | 0.09 |
| Median (IQR) C-tot 6-months | 182 (156-222) | 202 (160-213) | 0.60 |
| Mean (SD) C-tot 12-months | 191.58 (51.0) | 207.2 (24.7) | 0.30 |

Table 3S. Changes in anthropometric values and lipid profile according to gender

|  | **Female (n= 11)** | **Male (n= 18)** | **p-value** |
| --- | --- | --- | --- |
| Mean (SD) weight baseline | 62.2 (8.5) | 78.8 (11.4) | 0.0003 |
| Mean (SD) weight 6-months | 62.3 (8.8) | 78.3 (11.8) | 0.001 |
| Mean (SD) weight 12-months | 63.3 (8.8) | 79.6 (11.8) | 0.001 |
| Median (IQR BMI baseline | 24.6 (21.7-28.4) | 24 (21.7-29.4) | 0.16 |
| Median (IQR BMI 6-months | 25.8 (23.9-28.3) | 25.7 (23.5-28.7) | 0.26 |
| Mean (SD) BMI 12-months | 25 (3.3) | 24.4 (3.5) | 0.29 |
| Mean (SD) flank baseline | 94 (10.5) | 96.2 (5.7) | 0.46 |
| Mean (SD) flank 6-months | 94.2 (6.9) | 94.1 (5.4) | 0.96 |
| Median (IQR) flank 12-months | 90 (89-97) | 95 (92-99) | 0.12 |
| Mean (SD) abdomen baseline | 85.6 (10.8) | 93.3 (9.3) | 0.05 |
| Mean (SD) abdomen 6-months | 86.2 (11.7) | 92.9 (8.8) | 0.09 |
| Mean (SD) abdomen 12-months | 85.7 (11.4) | 93.4 (8.7) | 0.06 |
| Mean (SD) LDL baseline | 119.9 (16.3) | 104.1 (30.4) | 0.13 |
| Mean (SD) LDL 6-months | 117.3 (30.0) | 106.3 (35.9) | 0.40 |
| Mean (SD) LDL 12-months | 128.1 (27.7) | 116.3 (35.1) | 0.36 |
| Mean (SD) HDL baseline | 54.9 (14.8) | 49.4 (11.2) | 0.26 |
| Mean (SD) HDL 6-months | 61.7 (14.8) | 51.9 (12.6) | 0.07 |
| Median (IQR) HDL 12-months | 68 (60-70) | 53 (44-57) | **0.03** |
| Median (IQR) C-tot baseline | 202 (183-209) | 188 (149-197) | 0.10 |
| Median (SD) C-tot 6-months | 200.7 (32.1) | 183.2 (39.9) | 0.23 |
| Median (IQR) C-tot 12-months | 205 (199-234) | 209 (171-225) | 0.38 |

Table S4. Lipd profile changes in people without dyslipidemia (N=7)

| **Variables** | **Baseline VS 6 months**  **Median (IQR)** | | **p-value** | **Baseline VS 12 months**  **Median (IQR)** | | **p-value** |
| --- | --- | --- | --- | --- | --- | --- |
| Total cholesterol (mg/dL) | 188 (175–197) | 199 (176–214) | 0.297 | 188 (175–197) | 209 (203-226) | 0.016 |
| LDL (mg/dL) | 108(95–114) | 123 (97–125) | 0.297 | 108 (95–114) | 120 (104-138) | 0.016 |
| HDL (mg/dL) | 59 (47–63) | 65 (49–68) | 0.343 | 59 (47–63) | 62 (48–71) | 0.250 |
| Triglycerides (mg/dL) | 88 (58–178) | 77 (66-254) | 0.469 | 88 (58–178) | 76 (71-277) | 0.821 |
| Total cholesterol/HDL | 3.19 (2.77-3.79) | 3.15 (2.70-4.61) | 0.812 | 3.19 (2.77-3.79) | 3.41 (2.77-4.81) | 0.687 |
| Score2-2OP* | 3.95 (2.8-6.8) | 3.6 (2.7-5.4) | 0.094 | 3.95 (2.8-6.8) | 3.65 (2.8-4.6) | 0.312 |
| *Data on 6 people | | | | | | |

Table S5. Lipid profile changes in people with dyslipidemia (N=22)

| **Variables** | **Baseline VS 6 months**  **Median (IQR)** | | **p-value** | | | **Baseline VS 12 months**  **Median (IQR)** | | | **p-value** |
| --- | --- | --- | --- | --- | --- | --- | --- | --- | --- |
| Total cholesterol (mg/dL) | 190.5 (176-207) | 200.5 (156-212) | | 0.539 | 190.5 (176-207) | | 199 (171-229) | 0.033 | |
| LDL (mg/dL) | 119.5 (101-129) | 112 (77-141) | | 0.616 | 119.5 (101-129) | | 124 (95-151) | 0.012 | |
| HDL (mg/dL) | 49 (40-62) | 51 (46-64) | | 0.014 | 49 (40-62) | | 55 (44-68) | 0.016 | |
| Triglycerides (mg/dL) | 98.5 (75-121) | 90 (62-135) | | 0.780 | 98.5 (75-121) | | 88 (68-131) | 0.246 | |
| Total cholesterol/HDL | 3.53 (3.29-4.06) | 3.33 (2.80-4.17) | | 0.074 | 3.53 (3.29-4.06) | | 3.34 (3.13-4.01) | 0.288 | |
| Score2-2OP* | 5.25 (4.1-7.3) | 5.2 (3.9-7.3) | | 0.856 | 5.25 (4.1-7.3) | | 4.3 (3.1-6.6) | 0.300 | |
| *Data on 17 people | | | | | | | | | |

Figure 1S. Percentage of people with HIV who reported at least one adverse event.

Figure 2S. Percentage of people with HIV who reported Injection Site Reactions.

Figure 3S. Percentage of people with HIV who reported the nodule on the injection site and muscular pain. RPV: Rilpivirine; CAB: Cabotegravir

Figure 4S. Percentage of people with HIV who reported fever, fatigue or gastrointestinal disorders.

Figure 5S. Percentages of people with HIV who reported nervous adverse events.

Table S6. Changes in QoL score at baseline, 6 Months and 12 months.

| **Variables** | | **Baseline VS 6 months**  **Median (IQR)** | | | **p-value** | | **Baseline VS 12 months**  **Median (IQR)** | | | **p-value** |
| --- | --- | --- | --- | --- | --- | --- | --- | --- | --- | --- |
| QoL score | 60 (57-65) | | 65 (64-65) | <0.001 | | 60 (57-65) | | 66 (65-66) | <0.001 | |
